# Great cormorants and grey herons depredating at finfish aquaculture: Factors affecting the human–wildlife conflict

**DOI:** 10.1007/s13280-025-02218-5

**Published:** 2025-07-24

**Authors:** Camilla Ekblad, Mats Westerbom, Toni Laaksonen, Markus Kankainen, Antti Ovaskainen, Suvi Sinisalo, Veijo Jormalainen

**Affiliations:** 1 Natural Resources Institute Finland, Latokartanonkaari 9, 00790 Helsinki, Finland; 2 Department of Biology, University of Turku, 20014 Turku, Finland; 3 Natural Resources Institute Finland, Itäinen Pitkäkatu 4 A, 20520 Turku, Finland

**Keywords:** *Ardea cinerea*, Foraging, Nocturnal, *Phalacrocorax carbo sinensis*, Prey size

## Abstract

Sustainable aquaculture requires consideration of its interactions with wildlife. Human–wildlife conflicts arise when piscivorous birds, such as cormorants and herons, depredate in fish farms. By surveillance cameras, we quantified the depredation pressure of birds at fish farms along the Finnish coast. The pressure varied considerably between farms, from no bird visits to daily losses of hundreds of fish. Grey herons were most numerous and depredated 2–5 times more fish than cormorants, which are commonly regarded bigger threats. Depredation rates decreased with the increasing fish size even though cormorants also took large fish. Piscivorous raptors seldom foraged in farms but were interested in larger fish. Proximity to bird breeding colonies did not affect the predation pressure. Protective nets were effective against raptors and cormorants, but grey herons used them as depredation platforms. Knowledge on factors explaining depredation rates is valuable for science-based planning of measures to mitigate the human–wildlife conflict.

**Supplementary Information:**

The online version contains supplementary material available at 10.1007/s13280-025-02218-5.

## Introduction

Aquaculture is a rapidly growing, global industry and a significant source of animal protein for the expanding human population (Garlock et al. 2020; FAO 2022). Over the past 30 years, the global volume of aquaculture farming has multiplied with an average growth rate of nearly 7% per annum (FAO 2022). This growth has placed increasing pressure on natural ecosystems, leading to adverse interactions between wildlife and aquaculture producers (Callier et al. 2017; Carballeira Braña et al. 2021). Many wild animals, such as pinnipeds, cetaceans, and seabirds, that typically feed on wild fish stocks, are often attracted to marine finfish aquaculture (Callier et al. 2017). Economic losses to finfish aquaculture and fisheries caused by piscivorous seabirds have increased over the past decades in Europe, coinciding with the growth of the industry and the simultaneous strengthening of some protected bird populations (Cowx 2013; Marzano et al. 2013; Carss 2022). In addition to the direct consumption of farmed fish, avian predators cause economic losses by injuring fish (Kortan et al 2008; Westerbom et al. 2025) and by inducing stress, which may make fish more susceptible to diseases or reduce their growth (Cowx 2013).

The rapid expansion and population growth of great cormorant (*Phalacrocorax carbo sinensis*, hereafter cormorant) in Europe have led to widespread human-wildlife conflicts, resulting in socioeconomic, ecological and conservation impacts (Carss and Marzano 2005; Bregnballe et al. 2015; Carss 2022). With a current European population size approaching 0.8–1 M adult individuals (BirdLife International 2024), the effects of cormorants on fisheries and aquaculture can potentially be substantial. There is, however, considerable uncertainty regarding the magnitude of this impact (Ovegård et al. 2021), also raising indecision on how to deal with the growing cormorant population (Marzano et al. 2013). The ‘cormorant problem’ is particularly challenging to solve due to fundamental differences in stakeholder opinions (Carss 2022; Saarikoski et al. 2024) and a lack of quantitative data on their effects (Marzano et al. 2013; Tixier et al. 2021). Therefore, scientific knowledge regarding the factors influencing the magnitude of human–wildlife conflicts is crucial for planning effective management actions.

In Finland, the cormorant population has increased from the first few breeding pairs in 1996 to nearly 32 000 breeding pairs in 2024. Today, their distribution spans the entire Finnish coast (Rusanen 2024). Cormorants are, however, not the sole avian piscivorous foragers on Baltic Sea finfish farms. The grey heron (*Ardea cinerea*) population has been growing and has caused significant fish losses at some fish farms (Manikowska-Ślepowrońska et al. 2016). In Finland, grey herons primarily breed along the southern coast. The population began to increase in the 1990s and had reached 1000–1500 pairs by 2018 (Lehikoinen et al. 2019; Luomus 2024). Both cormorants and grey herons are generalist gregarious piscivorous birds that feed on a variety of fish of different sizes (Gwiazda and Amirowicz 2006; Lehikoinen et al. 2011). As highly flexible competitors for the same resources, generalist predators are often involved in human–wildlife conflicts (Dehnhard et al. 2021).

Despite significant concerns regarding the impact of cormorants and grey herons on sea-cage finfish farms, there is surprisingly little quantitative and systematic evidence regarding the frequency and magnitude of depredation (Marzano et al. 2013; Tixier et al. 2021). This particularly concerns the northern Baltic Sea, where interactions between avian predators and sea-cage finfish aquaculture have not been quantified, despite an awareness of the potential problem. Additionally, there is a lack of research assessing the effectiveness of various measures to mitigate the impacts caused by the two species (Cowx 2013). Management efforts aimed at reducing the potential impacts of depredation, disease transmission, and fish stress require a better understanding of the factors influencing cormorant and grey heron distribution patterns, as well as their feeding behaviour across aquaculture farms.

Here, we present quantitative data on foraging pressure and feeding behaviour of cormorants and grey herons when they depredate at fish farms along the Finnish coast. Our study aimed to identify which species foraged at the cages, factors influencing their visiting and foraging intensity, and the effectiveness of the protection measures implemented by the farmers. We hypothesized that the abundance of the bird species at fish farms and the extent of depredation would be related to (1) the size and distance to their nearest breeding colony, (2) the body size of fish cultured at distinct aquaculture farms, and (3) the type of protection measures taken at each facility. We predicted that fish consumption by cormorants and grey herons at aquaculture facilities would increase with the proximity and size of nearby bird breeding colonies and that both species primarily would depredate fingerling (< 20 cm) fish. Based on common perceptions and large differences in the regional population sizes between the bird species, we also predicted that overall depredation losses would be more attributable to cormorants than to grey herons. The results of this study can help identify factors affecting predation pressure, define the magnitude and share of the direct impact caused by the two bird species, and provide management with tools to mitigate this impact.

## Materials and methods

### Data collection and camera surveillance

**Fig. 1:**
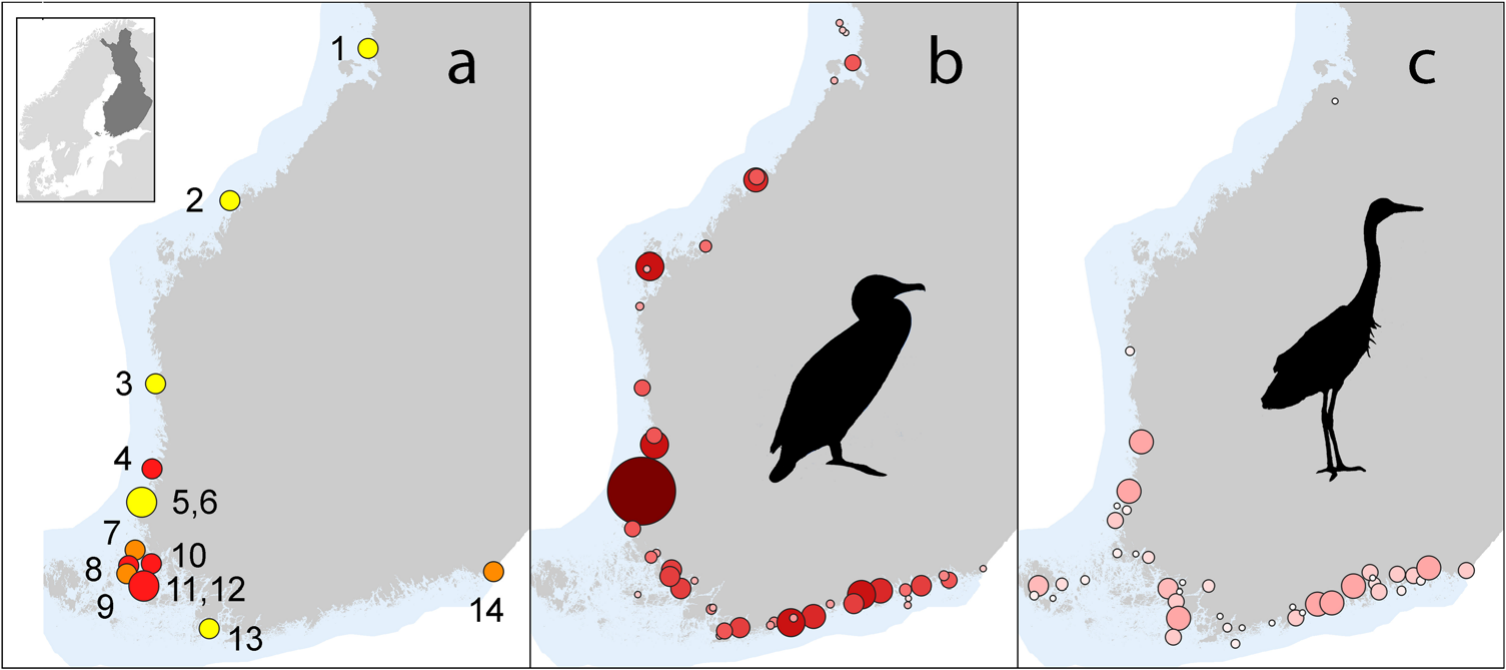
**a** The locations of the monitored fish farms. Red dots show farms monitored only in 2022, orange dots farms monitored both in 2022 and 2023, and yellow dots farms monitored only in 2023. The larger dots represent two distinct cages monitored at the same farm, 50–500 m apart. The individual cages are referred to with the same numbers in all figures throughout the manuscript. Location and size of **b** cormorant (8–2000, max 6800 pairs) and **c** heron (1–75 pairs) breeding colonies along the Finnish coast. Larger dots with darker colours represent higher numbers of birds. The colour shades but not the dot sizes are comparable across the maps

In 2022 and 2023, camera surveillance was implemented in cooperation with ten fish farmers at 13 fish farms along most of the Finnish coastline. The farms were selected based on interviews, and participation was voluntary. Consequently, farmers experiencing problems may be overrepresented in the data, as these entrepreneurs might have been more interested in participating. Three farms were monitored in both years, and at two farms, two fish cages were monitored simultaneously (Fig. 1). In total, 17 fish cages were monitored; eight in 2022 and nine in 2023. In two cages (the same farm in two consecutive years; denoted as 9a and 9b), the fish species was whitefish (*Coregonus lavaretus*), while rainbow trout (*Oncorhynchus mykiss)* was farmed in all other cages. The monitoring of the fish farms was conducted using remotely monitored, stand-alone solar-powered video surveillance cameras (Dahua DH-SD22204UE-GN, PTZ, 2 Mpx 4x (2.7–11 mm), 12*–*28 fps, 1280 × 720 /920 × 1080 resolution) installed on the fish cages. A small percentage of the cages were not visible in the recordings. Details regarding the cameras and example views are provided in Online Resource S1. Video footage was recorded in 1-h intervals and transmitted over a 4G mobile network to a server, covering various periods from May to October. In 2022, the video was set to record from 04 to 22 in September and 06–20:30 in October to conserve energy. In total, 1320 days of footage were recorded, averaging 78 (37–155) days at each facility (Online Resource S2).

### Analyses of video recordings

The surveillance cameras recorded > 26 000-h video footage, equivalent to 16 man-years of work if analysed at normal speed. Hence, we subsampled our data and analysed every 6th day, to avoid possible weekday induced patterns. The recordings were analysed manually at 0.5–32 times recording speed. Whenever there was bird activity, the video was rewound and watched at 0.5–3 times speed to quantify the number of birds present, their depredation attempts and the number of successful attempts.

From the video recordings, we extracted the following information (**bolded text**) for each hour for grey herons, cormorants, ospreys, and white-tailed eagles.

A) **Occurrence**. (1) The **maximum number and species of birds potentially foraging** at the cage, i.e. herons standing on the net, cormorants swimming in the cage and raptors flying close over. (2) The **time spent at the cage**. For herons, we counted the number of individuals standing on the net at 5-minute intervals, and calculated the mean number of herons per hour, further refined to ‘mean number of herons per hour per day’ by calculating the average of the hourly means. **Heron minutes** at cages per day was calculated as the mean number of herons per hour per day × 60 min × 24 h and rounded to whole numbers. For cormorants, we recorded the time in minutes that the birds were swimming in the cage or resting on the inner constructions. If multiple birds were present at the same time, the minutes were multiplied with the number of birds. **Cormorant minutes** per day were then calculated by summing the minutes of each hour.

B) **Depredation**. Cormorant depredation generally comprised a cormorant entering the cage where it swam and dove continuously until it left the cage, with or without surfacing with and eating one or several fish. The number of (3) **depredation attempts** was quantified as a peck for grey herons, a dive for cormorants and for raptors dipping the feet in the water. A depredation attempt was considered successful, if the bird caught a fish, and recorded as (4) **depredated fish**. Of these, we further distinguished the events, where a caught fish was dropped or escaped.

Finally, we recorded weather conditions for each hour, and in addition, the person (*n* = 8) analysing the hour made an assessment of the watching conditions (good, regular, poor, very poor).

### Possible biases and means to deal with them

We found that grey herons foraged actively also during nights. To account for the few dark hours when bird behaviour could not be recorded (mainly in late summer), we corrected the predated numbers of fish by grey herons per day to [number of predated fish in one day]/[number of hours analysed] × 24. On average, 21.3 h were analysed on days when grey herons were present, and on average 21% additional fish were added.

Harsh weather and poor visibility sometimes complicated the interpretation. Therefore, if the watching conditions on the sampling day were inadequate, we analysed the closest day with sufficient conditions instead. Some depredation attempts or depredated fish might have gone unnoticed if they occurred in the small part of the cages that fell outside the frame, although typically, the birds were only temporarily in a non-visible part. In most cages, the fishes were so large that the cormorants highly unlikely were able to swallow them while diving. If the cormorant had swallowed a smaller fish before surfacing, its throat was visibly swollen, which was assigned as a depredated fish. However, it cannot be ruled out that occasionally more than one fish was swallowed during a dive in the cage containing smaller fish. Overall, these effects are small, but the number of depredated fish and attempts may be slightly underestimated.

### Explanatory variables

#### Proximity to breeding colonies

For each surveyed fish cage, we measured the distance to the nearest active cormorant- and heron colony in QGis. The bird colony data (location and number of breeding pairs in each year) was obtained for cormorants from the Finnish Environment Institute. No such data exist for grey herons; hence, we obtained observation data from BirdLife Finland by which we compiled a breeding distribution map. Since the number of breeding pairs did not affect the results, the number of pairs was excluded from the final models.

#### Fish size and species

Fish sizes were obtained from fish farms at the beginning and end of the farming period and in some cases also in between. The size estimates were obtained by calculating the growth factor between the given values. Due to the insufficient number of farms containing whitefish, fish species could not be included in the models.

#### Protective net

Of the 17 fish cages, 15 were covered with protective nets. One net was removed during the season. The mesh size varied between 50 and 150 mm, most falling within the range of 50–80 mm. The height of the frames varied between 60 and 120 cm. Some nets were elevated in the middle of the cage by a supportive structure or feeding device.

Unfortunately, we were not able to properly model the effect of the nets, as there were not enough duplicates of different sizes and heights. Furthermore, it turned out that also factors that could not be controlled, such as how well the net was fastened and tightened, played a crucial role concerning the depredation. Since there, however, seemed to be some clear effects, we will comment on these based on observations.

### Statistical analyses

To answer our research questions, we implemented several statistical models using R version 4.4.1 (R Core Team 2024). For the statistical modelling, we used generalized linear mixed models (GLMM) with negative binomial error distribution in the R package glmmTMB (Brooks et al. 2017). The response variables were bird minutes spent at the cage per day (*frequency of occurrence, hereafter occurrence*) or number of predated fish per day (*depredation*). Post hoc comparisons were performed with Tukey’s test in the package emmeans (Lenth 2023). The fish cage ID (*n* = 17) was included as a random factor in all models.

We first tested whether the (1) occurrence and (2) depredation differed between the bird species and whether they depended on the size of the fish. The explanatory variables in these models were the bird species and the fish size, and their interaction. (3) Occurrence and (4) depredation were thereafter modelled separately for grey herons and cormorants. The explanatory variables in these species-specific models were the month as a classification factor, and fish size, the distance to the nearest colony of the bird species and latitude as standardized continuous factors. The correlation between bird occurrence and depredation was modelled with depredation as dependent variable, occurrence as explanatory variable, and species and cage ID as random factors.

Further, we modelled (5) the daily variation in foraging intensity separately for grey herons and cormorants. Here, we counted the hourly occurrence instead of the daily occurrence (the average number of minutes that the birds were present at each hour of the day for each cage), using only the days where the species was visiting the cage (*n* cages = 10 for grey herons and 7 for cormorants). The response variable was the hourly occurrence, and the explanatory variable was the hour (as a factor variable). All models are presented in Online Resource S3.

### Legislation on protected birds

Both cormorants and grey herons are protected under the Nature Conservation Act (9/2023) and the EU Birds Directive (Directive 2009/147/EC). Article 9 of the directive permits derogations under specific conditions, for which permits in Finland can be applied for from the Centres for Economic Development, Transport and the Environment (ELY). Here, birds are generally not shot at fish farms.

## Results

### Bird occurrence at fish cages

**Fig. 2:**
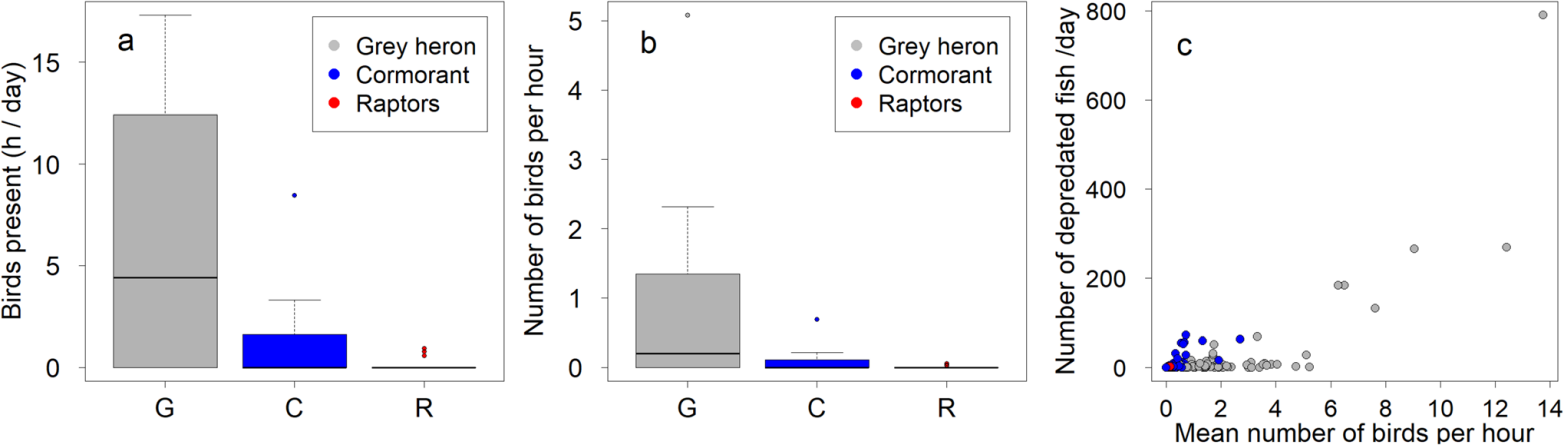
Distribution of **a** hours when grey herons, cormorants, and raptors were present at fish cages, and of **b** the mean of the maximum number of potentially foraging birds present at cages per hour, cage average. In the boxplots, the coloured box indicates the 50% quartiles, the thick line shows the median, and the thin lines above the boxes show the 1.5 × interquartile range. Possible outliers are shown as points. **c** The number of depredated fish per day in relation to the number of birds present at the cage (daily mean of the maximum number of birds present at the cage per hour)

The bird species most frequently occurring at the fish cages was grey heron (at 10 of the 17 cages; 59%), followed by cormorants (7 cages; 41%). Raptors (osprey *Pandion haliaetus* and white-tailed eagle *Haliaeetus albicilla* combined as raptors) were detected foraging on fish a few times at three cages (18%). Gulls (Larinae) occurred frequently at the fish cages, but they almost exclusively foraged on already dead fish and were not considered further. In three cages, no birds were foraging. Grey herons occurred most frequently (Fig. 2A) and in greater numbers (Fig. 2B) at the cages than cormorants and raptors (Table 1). The highest number of grey herons foraging simultaneously at a cage was 39. The number of predated fish increased significantly with the number of birds present (*z* = 6.21, *p* < 0.0001), although especially grey herons were often present without foraging (Fig. 2C).

**Table 1:** Parameter estimates, Wald Z statistics (z), and probabilities (*p*) for models explaining variation in the frequency of occurrence and depredation of birds at fish cages along the Finnish coast. Estimates for species are given in relation to grey heron

	Occurrence (minutes per day)			Depredation (# of fish per day)		
	Estimate ± SE	z	*p*	Estimate ± SE	z	*p*
(Intercept)	7.55 ± 0.78	9.70	< 0.001	3.79 ± 0.74	5.15	< 0.001
Fish size (kg)	− 4.34 ± 0.65	− 6.69	< 0.001	− 7.59 ± 1.03	− 7.38	< 0.001
Species		17.56	< 0.001		10.26	< 0.001
Cormorant	− 9.00 ± 0.67			− 4.97 ± 0.62		
Raptors	− 12.04 ± 0.78			− 8.51 ± 0.87		
Species * Fish size		10.48	< 0.001		8.42	< 0.001
Cormorant	6.61 ± 0.69			− 7.08 ± 0.88		
Raptors	6.59 ± 0.70			− 8.06 ± 0.99		

### Numbers of fish depredated by birds

**Fig. 3:**
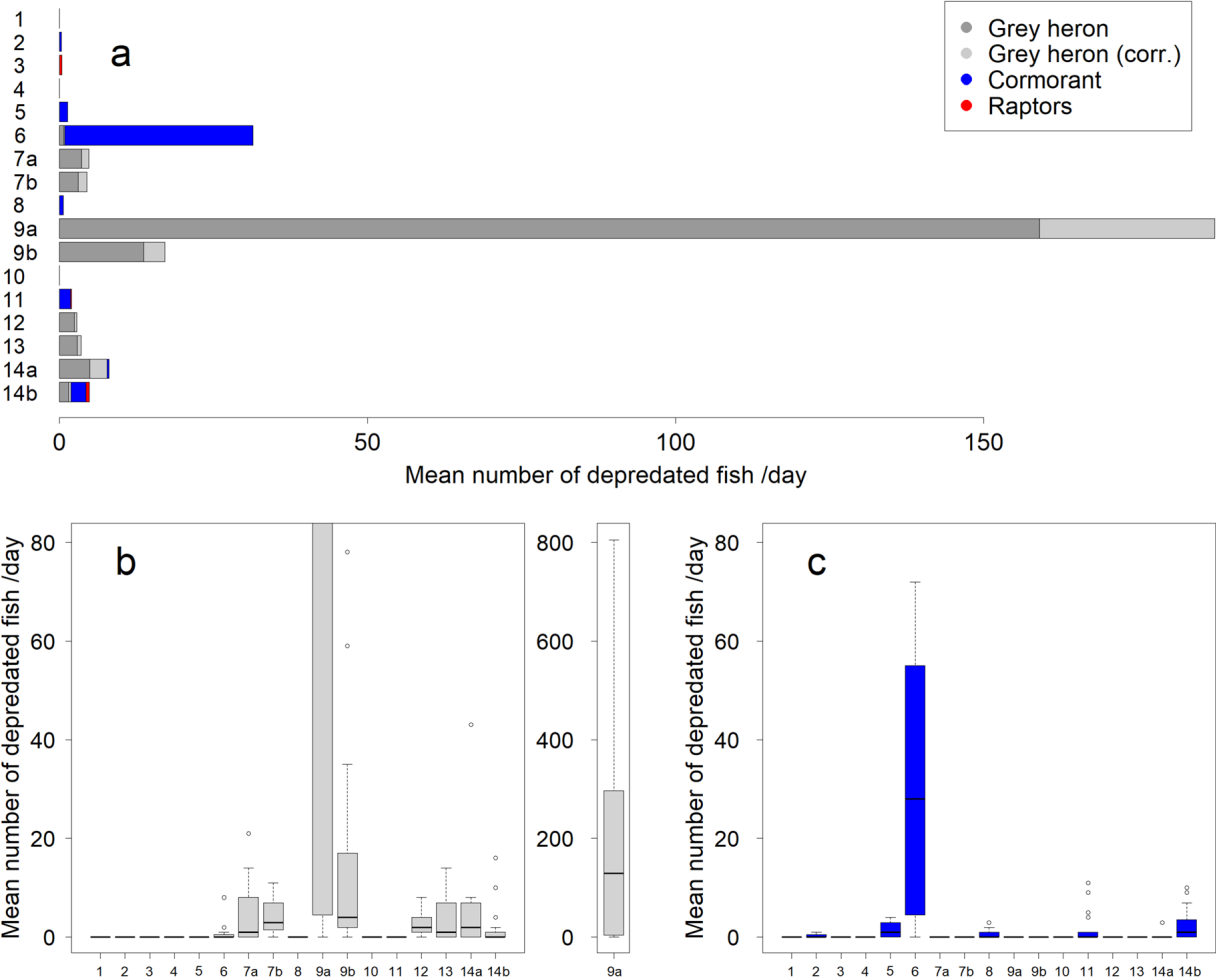
**a** The mean number of fish depredated by birds per day at 17 video surveyed fish cages along the Finnish coast. *Grey heron (corr.)* refers to additional depredation owing to the correction for the dark hours that could not be quantified with the video material. The distinct cages are plotted on the *y*-axis. The farms monitored in both years are annotated **a** for 2022 and **b** for 2023. Distribution of daily numbers of depredated fish by **b** grey herons (corr.) and **c** cormorants at the monitored cages (*x*-axis). The coloured boxes indicate the 50% quartiles, the thick lines the median, and the thin lines above the boxes the 1.5 × interquartile range. Possible outliers are shown as points. Cage 9a was subjected to a tenfold depredation by grey herons compared to the other cages and is shown separately with a different scale

The highest depredation was attributed to grey herons, followed by cormorants, while raptors depredated only a very small number of fish (Fig. 3, Table 1). There was a considerable variation in the number of fish depredated in the different cages (n analysed days = 224). The average number of fish depredated by all species per day over all cages was 13.5 (value corrected for the dark hours (‘corr’): 15.8), but it ranged between zero in three cages and a maximum of 159 (corr: 188) in one extreme cage, the median being 2.4 (corr.: 2.8) (Fig. 3A). On 46% of the days, no bird depredation was recorded. Also, the daily variation in depredation within the same fish cage was substantial (Fig. 3B, C). The highest recorded number of predated fish in a cage in a single day was 790 (corr.: 807).

### The occurrence of different bird species in relation to fish size

**Fig. 4:**
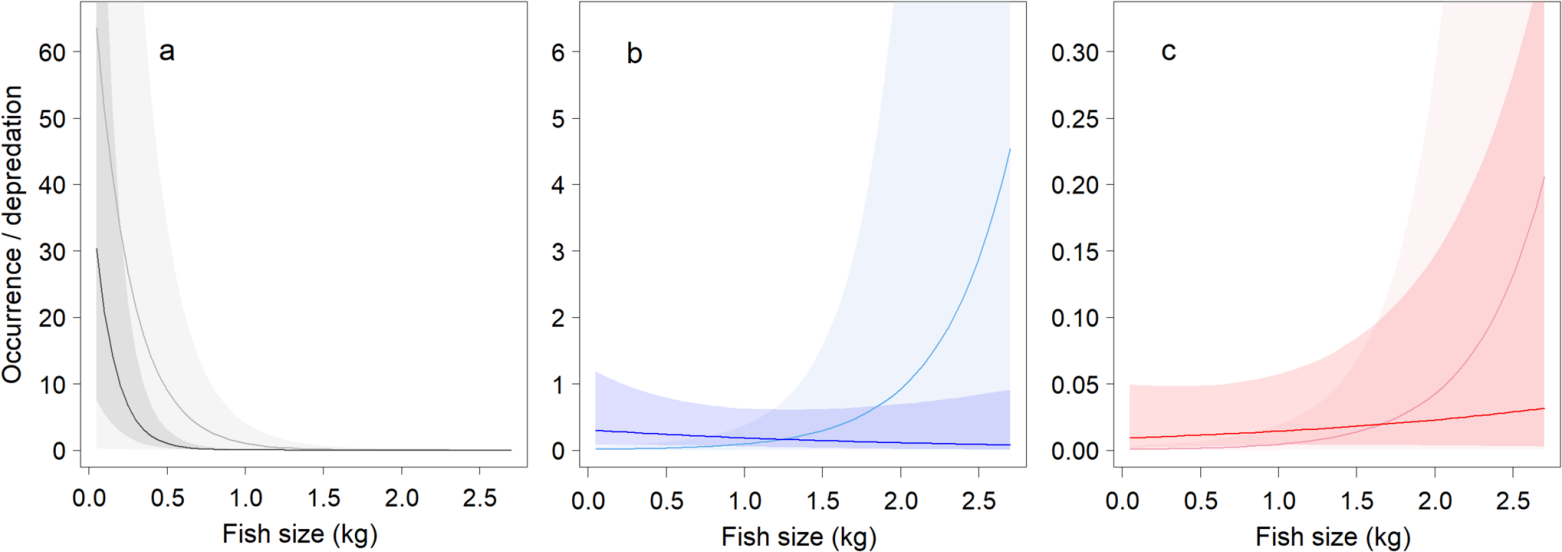
Modelled frequency of occurrence of **a** grey heron, **b** cormorant and **c** raptors (minutes that birds are present per *hour*, light lines) and the number of depredated fish per *day* (dark lines) at fish cages in relation to the size of the farmed fish. The shaded areas indicate 95% confidence intervals. Note the different scales at the Y-axes in the three panels

Fish size was the most important predictor for the frequency of occurrence of different bird species (Fig. 4, Table 1). Grey herons occurred more frequently at cages with small fish, while cormorants and raptors occurred more frequently at cages with larger fish (Fig. 4) as indicated by the significant bird species-by-fish size interaction (Table 1). Grey herons consumed predominantly small fish, and although they occurred at cages with up to 1 kg fish, the depredation virtually ended at fish size of 0.5 kg (Fig. 4A). Even though cormorants spent more time at cages with larger fish, they predated more on smaller fish (Fig. 4B). Raptors occurred only at cages with fish larger than 0.85 kg, more frequently and with higher numbers of predated fish the larger the fish in the cage were (Fig. 4C).

### Temporal differences in bird occurrence

**Fig. 5:**
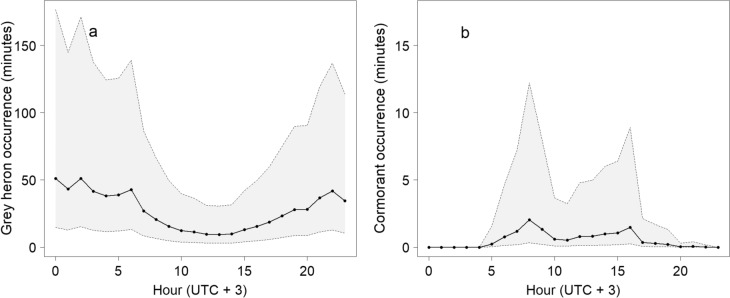
Daily occurrence of **a** grey herons and **b** cormorants at fish cages along the Finnish coast. The panels show the modelled number of minutes that grey herons (**a**) and cormorants (**b**) spent potentially foraging at depredated cages in each hour of the day. The shaded areas show the 95% confidence intervals

The grey herons and cormorants occurred at the fish cages at different times of the day (Fig. 5). The grey herons visited the cages most actively at night and less during the day (8–18). Contrary, the cormorants were day active and did not visit the fish cages at all in the night between 23 and 05. The cormorant foraging had two peaks, the highest in the mornings between 8 and 10 and another in the afternoons between 13 and 17 (Fig. 5). White-tailed eagles were present and made foraging attempts at the fish cages between 03 and 22, and ospreys between 05 and 20.

### Determinants of depredation

In the species-specific models, for grey herons both occurrence and depredation increased with decreasing fish size and towards the south (Table 2). There was an indication that grey herons occurred more frequently at cages located closer to the colonies (*p* < 0.1), while there was no effect of the colony proximity on the number of predated fish. There were no statistically significant differences in the heron occurrence between months. However, grey herons predated more fish in June and July than in other months (Table 2).

**Table 2:** Parameter estimates, Wald statistics (z), and significance (*p*) for models explaining variation in the frequency of occurrence and depredation of grey herons respective cormorants at fish cages along the Finnish coast in terms of month and distance to bird colonies. Temporal estimates are given in relation to month May

Grey heron	(a) Occurrence (minutes per day)			(b) Depredation (# of fish per day)		
	Estimate ± SE	z	*p*	Estimate ± SE	z	*p*
(Intercept)	− 0.06 ± 1.57	− 0.04	0.97	− 5.44 ± 1.81	− 3.00	0.003**
Fish size	− 3.07 ± 0.57	− 5.34	< 0.001***	− 4.34 ± 0.65	− 4.25	< 0.001***
Distance to colony	− 3.12 ± 1.89	− 1.65	0.10	− 1.14 ± 1.45	− 0.79	0.43
Latitude	− 7.08 ± 1.82	− 3.28	0.001**	− 4.76 ± 1.82	− 2.61	0.009**
Month		2.86	0.15		3.57	0.026*
June	0.11 ± 0.76			2.19 ± 0.88		
July	− 0.14 ± 0.84			2.03 ± 0.99		
August	0.36 ± 0.92			1.78 ± 1.05		
September	0.04 ± 1.00			1.37 ± 1.16		
October	− 1.98 ± 1.28			− 0.92 ± 1.55		

Cormorant	(c) Occurrence (minutes per day)			(d) Depredation (# of fish per day)		
	Estimate ± SE	z	*p*	Estimate ± SE	z	*p*
(Intercept)	− 13.05 ± 3.21	− 4.06	< 0.001***	9.58 ± 5.36	− 1.70	0.089
Fish size	0.20 ± 1.04	− 0.19	0.85	− 0.73 ± 0.94	− 0.78	0.44
Distance to colony	0.20 ± 1.73	0.12	0.91	− 0.16 ± 1.48	− 0.11	0.92
Latitude	0.20 ± 1.21	0.17	0.87	0.00 ± 1.15	0.00	0.99
Month		4.35	0.003**		5.18	< 0.001***
June	3.73 ± 1.25			3.03 ± 1.02		
July	4.47 ± 1.22			3.57 ± 1.03		
August	3.49 ± 1.45			1.91 ± 1.19		
September	5.23 ± 1.86			3.20 ± 1.60		

In the model for cormorants, fish size, proximity to the colony and latitude, had no effect on the occurrence of, or depredation by, cormorants. The cormorant occurrence and depredation at the cages peaked in June, and again in September, and collapsed in October (Table 2).

### Protective nets

**Fig. 6:**
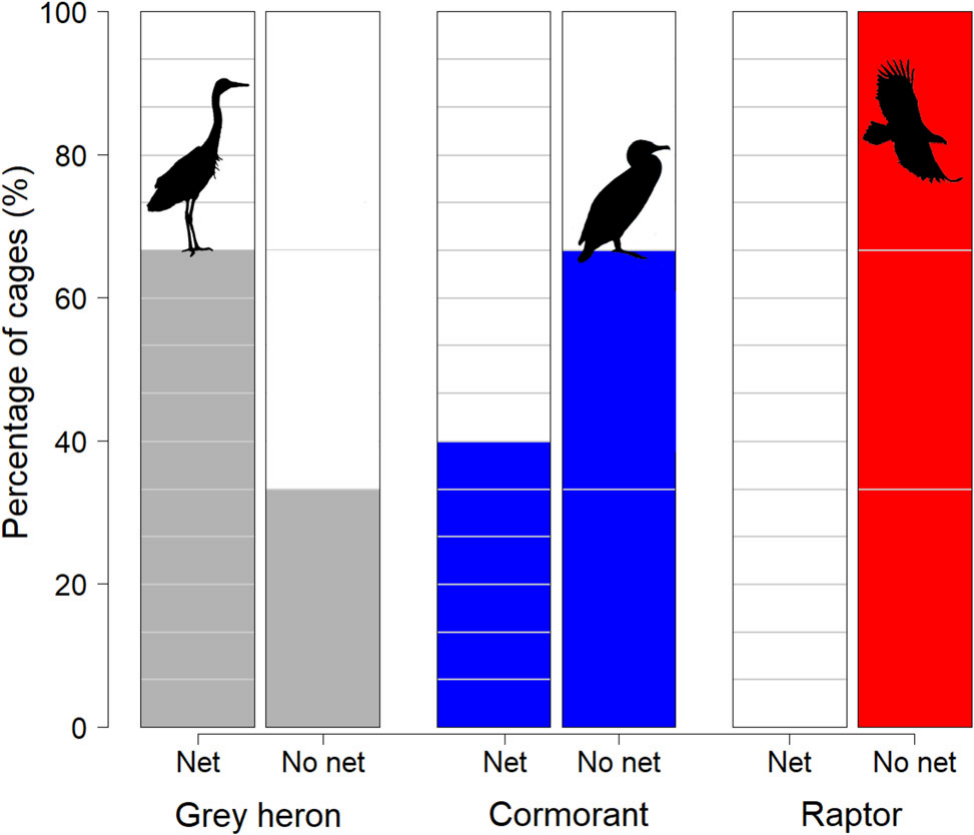
The depredation attempts of grey herons, cormorants, and raptors at fish cages in regard to protective nets. The coloured bars show the percentage of surveyed cages, at which the birds were making depredation attempts. Grey bar pairs represent grey herons, blue cormorants, and red raptors. The numbers of fish cages with a net, labelled “Net” (*n* = 15), and without a net, labelled “No net” (*n* = 3), are indicated by the blocks

Even though the role of protective nets could not be properly modelled due to insufficient numbers of unprotected cages, we were able to identify some factors that appeared to affect the numbers and depredation attempts of the birds. Observations are presented because they are relevant considering protection against these species. Raptors were foraging at all three netless cages but not at any protected cage (Fig. 6.). Herons, on the contrary, occurred almost exclusively at cages with nets, which they used as depredation platforms. The depredation was most severe when the distance to the surface was small, either because the net was low or loose. For cormorants, the role of the net was most complex. Cormorants depredated at 2/3 (66%) of the cages lacking net, and at 6/15 (40%) of the cages protected by a net (Fig. 6). Most fish, however, was depredated at a protected cage. The cormorants were able to climb into cages with nets with large mesh size, or between the structure and the net, if it was not well fastened.

## Discussion

Despite pan-European efforts to address the conflict between fisheries and cormorants, which have been ongoing since the end of the twentieth century (Carss and Marzano 2005; Marzano et al. 2013), quantitative data on the extent of depredation of aquaculture by birds remain limited. Here, we quantified, for the first time in the Northern Baltic Sea, the depredation pressure by piscivorous birds at fish cages along the Finnish coast. The frequency of occurrence and depredation by grey herons and cormorants at fish cages varied significantly among the fish farms. The distribution of the farm-specific averages in the number of depredated fish was strongly right-skewed, with a small median value (2.8 fish per day). The average number of depredated fish was several times larger than the median at corresponding farms. At a few farms, depredation losses were substantial. As there was a considerable variation in depredation pressure between different farms, we cannot provide exact numbers of total damage. But assuming a 150-day occurrence per year on the fish cages for both these two migratory bird species and using their average daily depredation rates (13.5 fish per day for grey heron, and 2.2 fish per day for cormorants), the yearly direct depredation losses would be on average 2025 and 330 fish per year per cage, respectively. These would represent 4.0% and 0.6% of an average-sized fish cage containing approximately 50 000 fish. These numbers do not include possible indirect losses, e.g. dead fish aggregating on the bottom of the cage.

Contrary to our predictions, grey herons depredated more fish than cormorants. Grey herons were present at fish cages in greater numbers during the night while cormorants were only present during day. This likely explains why cormorants are typically regarded as more common visitors at fish cages. The size of the farmed fish was the primary factor influencing the frequency of occurrence and depredation by different bird species. Grey herons primarily targeted small fish, while cormorants depredated fish of all sizes and raptors exclusively targeted large fish. Contrary to our predictions, the distance to the nearest breeding colonies of cormorants or grey herons did not affect their depredation pressure, although there was an indication of a relationship between depredation magnitude and the distance to the nearest grey heron colony.

### Bird presence at fish cages

With high densities of catchable fish, farms attract piscivorous birds (Díaz López 2017; Barrett et al. 2019). Cormorants and grey herons have been identified as the primary bird species causing conflicts at fishponds and fish cages in Europe (Kloskowski 2011; Manikowska-Ślepowrońska et al. 2016; Díaz Lopetz 2017; Adamek and Kajgrova 2022). However, the factors influencing the magnitude of depredation have been poorly understood. Although the population of cormorants breeding along the Finnish coast is 30*–*40 times larger than that of breeding grey herons, grey herons were more frequent visitors and caused greater fish losses than cormorants at the surveyed fish cages (see also Kloskowski 2011; Manikowska-Ślepowrońska et al. 2016). Grey herons frequently spent time at the fish cages also when they were not foraging, unlike cormorants. On average, grey herons depredated six times more fish than cormorants. In terms of biomass, however, the herons depredated just twice as much fish as the cormorants. Our study also revealed nocturnal behaviour among herons. Grey herons frequently foraged at night, a behaviour that has rarely been documented (but see Huang et al. 2024). This nocturnal activity is important to acknowledge, as depredation rates may otherwise remain largely underestimated. The morning and afternoon peaks in cormorant activity may be attributed to increased human disturbance around noon, when the farmers often visit the farms.

### Factors affecting bird depredation

The size of the fish was the most significant predictor of species frequency of occurrence and their depredation rate. Grey herons depredated only fish smaller than 500 g, and their depredation strongly targeted cages containing fish smaller than 100 g (median 85 g). Cormorants exploited a broader size range of fish. Although 80% of all depredated fish originated from a cage containing small fish (mean size 114 g), most of the remaining cages that were exploited contained relatively large fish with an estimated size of 600*–*2250 g. This size range exceeds the typical maximum reported range (up to 900 g) for cormorants (Čech et al. 2008; Steffens 2010) but fall within the maximum reported range (Belfethi and Moulai 2022). Both species were observed attempting to eat fish that were too large to swallow and had to be discarded by the bird. These fish apparently usually died, as they generally were left floating on the surface, thereby increasing depredation losses.

Cormorants primarily forage up to 25–30 km from their breeding colonies (Thaxter et al. 2012; Fijn et al. 2022), while grey herons may utilize feeding areas up to 20 km from their nests (Jakubas 2005). In the Czech Republic, higher densities of cormorants were recorded at fishponds located closer to breeding colonies (Adámek and Kajgrová 2022). At the cages we surveyed, the distance to the cormorant colonies did not influence the presence or depredation of cormorants, even though in the same area, the distance to and number of nests in cormorant colonies were associated with increased predation attempts in fyke nets (Westerbom et al 2025). Cormorants utilize extensive foraging patches and switch between them, regularly using adjacent areas as foraging grounds (Bugajski et al. 2013; Fijn et al. 2022). Consequently, the availability of other available prey influences the selection of foraging patches, and the profitability of the cages in this study as foraging sites was largely dependent on other factors than the core distance to a breeding colony. At the end of the season, cormorants leave the breeding colonies and aggregate in large flocks. The increased cormorant depredation observed in September compared to August was presumably due to the presence of these flocks prior to their southward winter migration. In Europe, where both resident, migrating and overwintering cormorants occur, the most intense depredation in aquaculture ponds has been attributed to non-resident birds (Seiche et al. 2012; Adámek and Kajgrová 2022).

### The role of protective nets on cages

Netting has been shown to be the most effective method for preventing or minimizing bird depredation at fish cages (Bildsøe et al. 1998). However, birds can, to some extent, learn to deal with the nets. Among the monitored cages, only three containing large fish (500–1800 g) lacked protective netting. Raptor depredation was recorded exclusively in these three open cages, indicating that nets effectively prevent raptors from foraging in the cages. Consequently, the raptors’ preference for the largest fish may be more apparent than real, as they only had access to large fish.

While open cages without protective nets encouraged cormorant depredation, this was not always the case. Despite suitable fish size, cormorants never visited one particular open cage, even though it was located just 4.4 km from a large, active colony. Telemetry data from GPS-tagged individuals from this colony (Lindén et al. unpublished) indicated that cormorants frequently visited the area, but they never visited the cage itself. Cormorant feeding behaviour is complex, and cormorants do not always depredate fish farms, even when these are close to active colonies, the fish size is appropriate for depredation and the cage is easily accessible.

In general, tightly fastened nets provided effective protection against cormorant depredation. Problems arose with nets that were loose and/or had a mesh size allowing intrusion into the cage through the mesh. When the mesh was small enough to prevent this but insufficiently fastened, the cormorants entered the cage between the frame and the net. At the farm experiencing the most severe predation, cormorants were even observed fishing through the loose net on windy days, when they were able to reach the surface at wave crests (Online Resource S1b). While we can only speculate about why this particular farm was so heavily impacted, it was the farm with small fish that had the highest net, and cormorants seemed to prefer having some space between the surface and the net. Additionally, the largest cormorant colony, with > 6000 pairs, was located 16 km away, and these cormorants may have exploited the farm.

Contrary to cormorants, grey herons foraged almost solely at cages with nets. They used the nets as platforms for foraging and pecked the fish through the mesh. The smaller the mesh size, and the closer the net was to the water surface, the better platform it was. Grey herons also entered cages through large mesh openings, or by pushing themselves between the frame and the net. When entering cages, they fished standing on the feeding automat or on the frame. The cage 9a, that experienced the highest level of grey heron depredation, was located in a sheltered archipelago, had a low frame and a net with small mesh size that was not elevated, making it particularly vulnerable to grey heron predation (Online Resource S1b). Here grey herons were foraging in harmony up to almost 40 at the time. This farm was monitored also in 2023 (9b), when it experienced significantly less depredation. In other cages, one or a few dominant grey herons chased away intruders, not only reducing the number of depredators but also limiting their own foraging time.

## Conclusions and recommendations

Surveillance camera monitoring of fish cages provided novel, quantitative data on depredation by piscivorous birds, enhancing our understanding of the variation in the magnitude of cormorant and grey heron depredation and the share of species involved. This knowledge is urgently needed to evaluate options for mitigating and managing conflicts (Tixier et al. 2021) and to explore possible compensation options for economic losses caused by these protected species. We observed significant variation in depredation pressure across fish farms. Nearly one-fifth of the surveyed cages had no bird problems at all and the median number of depredated fish over all cages was low, but in some cages the fish loss was substantial. The highest level of depredation was caused by grey herons. The size of the farmed fish was the most significant predictor of both the occurrence of and depredation by grey herons and piscivorous raptors, while cormorants showed interest in fish of all sizes. The distance to bird breeding colonies was not linked to increased depredation pressure, and even in the vicinity of breeding colonies, cormorants did not necessarily visit the fish cages. Therefore, proximity to a bird colony does not automatically result in higher bird presence and depredation at finfish aquaculture sites.

To be effective, the proper use of protective nets is crucial due to associated costs, which include additional production expenses and increased workload for fish farmers. The net should (1) be set tightly, as pots emerging in loose nets can serve as depredating platforms, (2) be elevated to prevent birds from reaching the water while standing on the net, (3) have a relatively small mesh size and (4) be properly fastened to the frame to prevent birds from entering the cage. Well-assembled nets provide good protection from depredating birds; however, bird responses to net types are species specific. Therefore, (5) the species composition of piscivorous birds in the area and the size of the fish in the cage is essential to consider when deciding on protective measures. Any potential governmental compensation for the production losses caused by protected bird species should primarily focus on minimizing the losses, e.g. compensating the costs arising from acquiring and maintaining the nets and structures necessary to deter birds from depredating from the aquaculture facilities.

## Supplementary Information

Below is the link to the electronic supplementary material.

Supplementary file1 (PDF 569 kb)

## Data Availability

The data is available at 10.1007/s13280-025-02218-5 (Ekblad et al. 2025).
