# Supplementary material for: Great cormorants and grey herons depredating at finfish aquaculture: Factors affecting the human–wildlife conflict
Source: Ambio. 2025 Jul 24;55(1):164–75. doi: 10.1007/s13280-025-02218-5 (PMC12672961; doi:10.1007/s13280-025-02218-5)
Supplement: Supplementary file 1 — Supplementary file1 (PDF 569 kb) [file 13280_2025_2218_MOESM1_ESM.pdf]

Supplementary Information: This Supplementary Information has not been peer reviewed.

Title: Great cormorants and grey herons depredating at finfish aquaculture: factors affecting the human-wildlife conflict.

Supplementary Material

Great cormorants and grey herons depredating at finfish aquaculture: factors affecting the human-wildlife conflict. Ambio. Ekblad C[1], Westerbom M, Laaksonen T, Kankainen M, Ovaskainen A, Sinisalo S, Jormalainen V.

[1] Corresponding author: Camilla Ekblad, Natural Resources Institute Finland, camilla.ekblad@luke.fi

Online Resource 1.

a) Details on the cameras and their set-up

In the study ten Dahua (DH-SD22204UE-GN) PTZ Network Cameras 2 Mpx 4x (2.7–11 mm) cameras were used to record the data. The cameras were set to record at 1280x720 resolution at 12 fps, aspect ratio 16:9 with a bitrate of 768 (CBR) in 2022. In 2023, the resolution was heightened to 1920x1080, but in one camera it had to be reversed to the lower because the network in the place was not sufficient to transfer the larger videos. In 2023, at locations with good connection quality, a higher framerate of 24 FPS or 28 FPS was tested. The recording used H. 264H video encoding. The recorded video was transferred over a mobile network (3G or 4G) via FTP into a remote server hosted by the University of Turku, where recordings were kept for later analysis. Dahua Network Speed Dome & PTZ Camera Web 3.0 was used to control the cameras, change the record and camera settings and to set up the remote server connection for video file transfer. Transferred files video format was DAV Video File (.dav). VLC media player was used for viewing and analysing the videos.

The factsheet for the camera can be found at https://www.dahuasecurity.com/asset/upload/uploads/soft/20201214/SD22204UE-GN_Datasheet_20201214.pdf (accessed 17.4.2025). Detailed descriptions about the specs, construction and assemblage of the camera systems are presented in Ovaskainen, A. 2024. Etähallittavan ja tallentavan kamerajärjestelmän kehittäminen ja käyttö lintulajien vierailun havainnointiin rannikon kalankasvatuslaitoksilla. MSc-thesis, University of Turku (in Finnish).

b) Snapshots from the surveillance cameras

On the following page views from some cameras are presented, showing the general watching view and some examples of depredation situations.

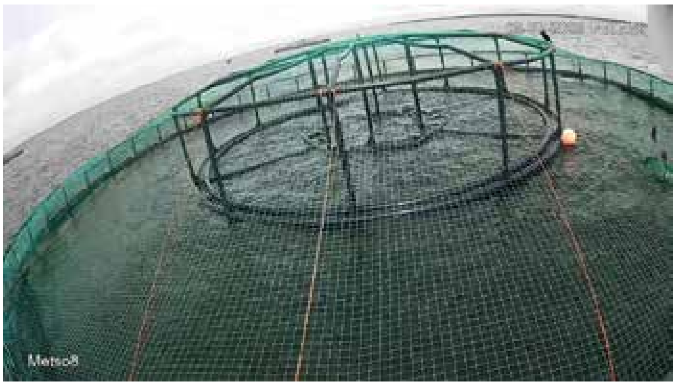

Cage 6 contained small fish and was subject to the most severe cormorant predation. The net was elevated, but the cormorants climbed in between the frame and the net, and at harsh weather they fished with their necks hanging through the net, as seen here. This behavior was seen only at this farm, at both monitored cages.

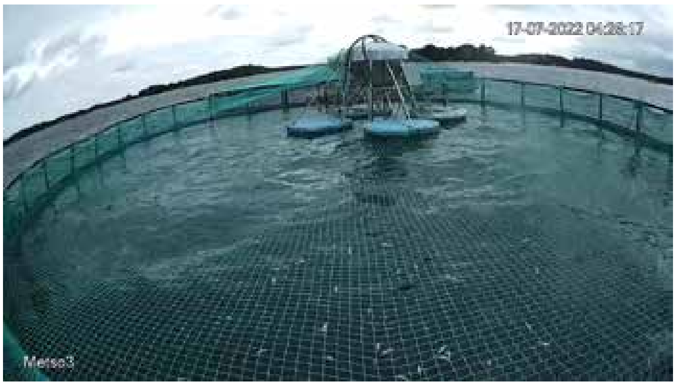

Cage 7a was elevated in the middle by the feeding device, but the herons were able to reach the water from lower parts. Here, dead fish are floating at the surface in early morning before cleaned away. The depredation differed at the cages in this farm, and the camera happened to be on a cage with relatively little heron depredation.

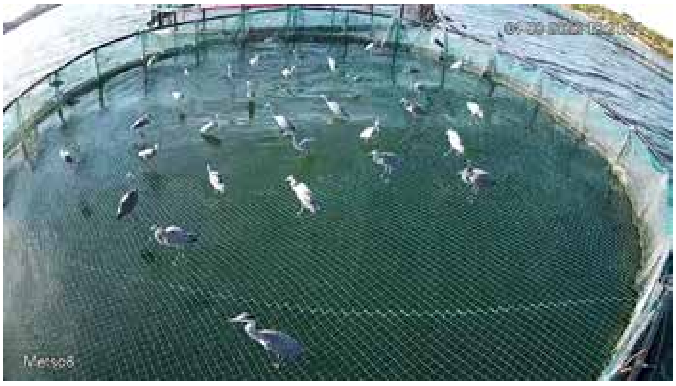

Cage 9a was subject to the most severe grey heron depredation. The mesh size is small, and the net is not elevated.

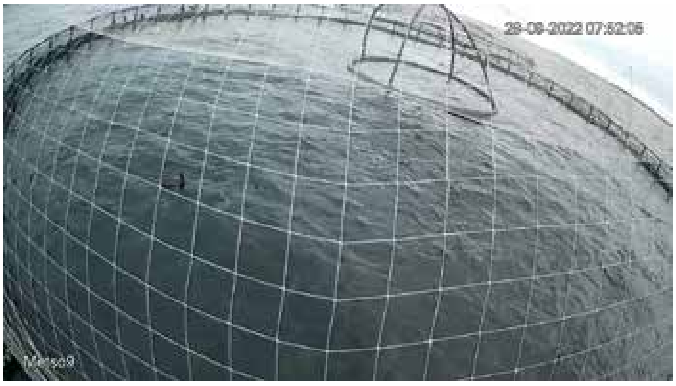

Cage 10 had an elevated net, but the mesh size was large, and the cormorants climbed in and out at the frame.

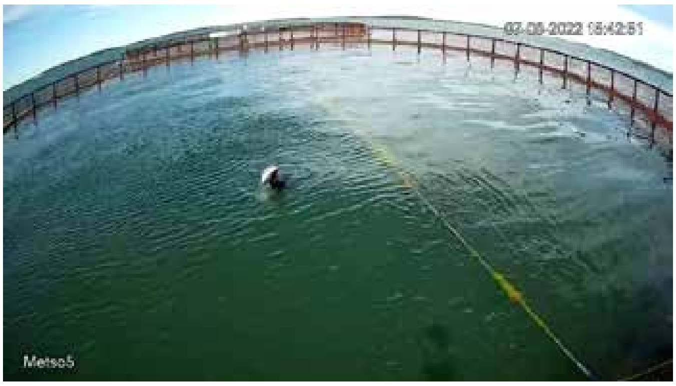

Cage 11 contained large fish and had no net. Here, the cormorants foraged frequently, and ate fish of considerable size, as seen in the picture.

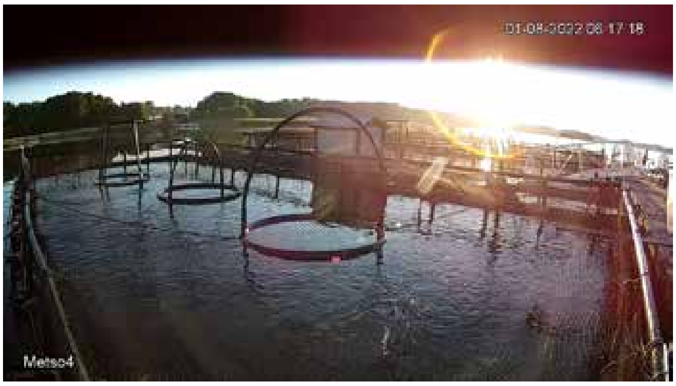

Cage 12 had structures elevating the net, but it was not tight in all places and the herons reached the water in pits. Here, a heron has caught a quite large fish.

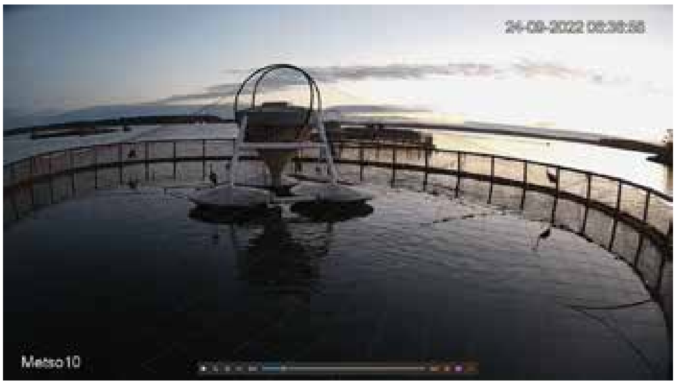

The net in cage 14a had large mesh size, and the herons climbed in and out of the cage. Here, the heron to the left very untypically dive head first into the water to catch fish. This behavior was seen only at this farm.

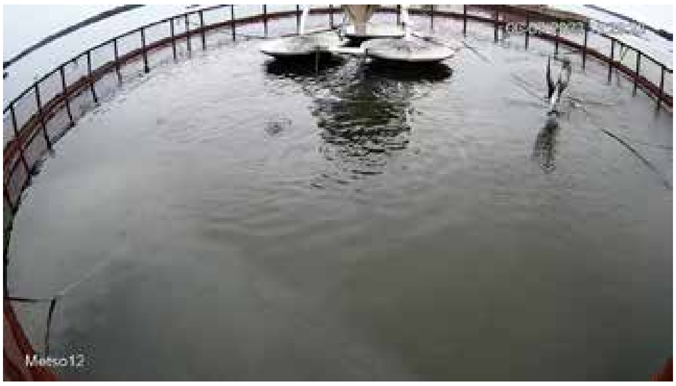

The net at cage 14b was removed mid-season, as the fish got entangled in it. Here, an osprey is cathing a fish. Also cormorants and herons depredated here.

Online Resource 2. Specifications of the monitored cages. The columns IV (April)–X (October) shows the time frame when each cage has been monitored, 2022 in dark and 2023 in light grey. ID = cage ID in Fig. 1A, Days = number of analysed days, Fish (g) = mean size of the fish during the monitoring period (in grams), Dist Pc = distance to the nearest cormorant colony (in km), Dist Ac = ditto for grey herons, Species = farmed fish species; Om = rainbow throat, Cl = whitefish, Net = cage has (+) or has not (-) a protective net.

ID	Year	Days	IV	V	VI	VII	VIII	IX	X	Fish (g)	Dist Pc	Dist Ac	Species	Net
1	2023	8								478	14,8	53,0	*Om*	+
2	2023	7								1220	43,4	164,0	*Om*	+
3	2023	19								1003	4,4	32,7	*Om*	-
4	2022	7								434	8,2	2,5	*Om*	+
5	2023	14								891	16,4	3,7	*Om*	+
6	2023	15								97	16,4	3,7	*Om*	+
7a	2022	17								65	23,3	26,0	*Om*	+
7b	2023	7								102	23,3	7,9	*Om*	+
8	2022	13								1066	25,3	23,6	*Om*	+
9a	2022	12								112	17,1	17,9	*Cl*	+
9b	2023	13								46	31,0	17,9	*Cl*	+
10	2022	9								2110	13,6	13,6	*Om*	+
11	2022	18								2436	15,5	31,2	*Om*	-
12	2022	18								393	16,8	34,3	*Om*	+
13	2023	13								124	24,1	11,9	*Om*	+
14a	2022	9								256	10,2	2,5	*Om*	+
14b	2023	27								1107	3,4	22,6	*Om*	+/-[1]

[1] The net was removed during the season (6.6.)

Online Resource 3. All models used in the manuscript are presented below. All models were run with the package glmmTMB (Brooks et al) in R version 4.4.1 (R Core Team 2024).

Variables and explanations

Occurrence:	Number of minutes, that birds spent at the cage *per day*
Occurrence.h:	Number of minutes, that birds spent at the cage *per hour*
	_Ac: Grey heron
	_Pc: Cormorants
Depredation:	Number of depredated fish per day
Species:	Grey heron (*Ardea cinerea*)
	Great cormorant (*Phalacrocorax carbo sinensis*)
	Raptors (White-tailed eagles *Haliaeetus albicilla* and Ospreys *Pandion haliaeetus*)
Month.f:	month (May-October) as a factor variable
Fish_size:	size of fish in kg
Distance:	distance to nearest colony of respective species; a) grey heron and b) cormorant in km
Lat:	Latitude (EUREF)
Cage_ID:	unique identifier for each monitored fish cage (n=17)
Hour.f:	hour of the day (0–23) as a factor variable

Models

1) Occurrence of bird species at the fish cages
   Occurrence ~ Species * Fish_size + (1|Cage_ID)

2) Depredation of bird species at the fish cages
   Depredation ~ Species * Fish_size + (1|Cage_ID)

3) Species-specific occurrence for a) grey herons and b) cormorants
   3a) Occurrence_Ac ~ Fish_size + distance_Ac + lat + month.f + (1|Cage_ID)
   3b) Occurrence_Pc ~ Fish_size + distance_Pc + lat + month.f + (1|Cage_ID)

4) Species-specific depredation for a) grey herons and b) cormorants
   4a) Depredation_Ac ~ Fish_size + distance_Ac + lat + month.f + (1|Cage_ID)
   4b) Depredation_Pc ~ Fish_size + distance_Pc + lat + month.f + (1|Cage_ID)

5) Daily variation in foraging intensity
   5a) Occurrence.h_Ac ~ hour.f + (1|Cage_ID), family = nbinom2
   5b) Occurrence.h_Pc ~ hour.f + (1|Cage_ID), family = nbinom2
